# Supplementary material for: The Requirements for Setting Up a Dedicated Structure for Adolescents and Young Adults with Cancer—A Systematic Review
Source: Curr Oncol. 2025 Feb 11;32(2):101. doi: 10.3390/curroncol32020101 (PMC11854605; doi:10.3390/curroncol32020101)
Supplement: Supplementary file 1 [file curroncol-32-00101-s001.zip › curroncol-3437138-supplementary.pdf]

Search strategy 20.01.2024 – Version 2 = 2367 hints

1. Cancer diagnoses	oncolog*[tiab] OR cancer[tiab] OR neoplasm*[tiab]
2. Different age categories	adolescen*[tiab] OR teenag*[tiab] OR "young adult*"[tiab] OR "young peopl*"[tiab] OR AYA[tiab] OR TYA[tiab] OR "Young Adult"[MeSH] OR Adolescent[MeSH] OR "teenager*"[tiab] OR AYAs[tiab]
3. Combine	#1 AND #2
4. Set up in hospitals / clinics	"model* of care"[tiab] OR "cancer service*"[tiab] OR "cancer care"[tiab] OR "standard* of care"[tiab] OR "practice guideline"[tiab] OR "position paper"[tiab] OR "clinical care"[tiab] OR "distinctive characteristics"[tiab] OR "program development"[tiab] OR "specialised service*"[tiab] OR "specialized service*"[tiab] OR "clinical program*"[tiab]
5. Combine	#3 AND #4
6. Humans only	animals[Mesh] NOT humans[Mesh]
7. Combine	#5 NOT #6
8. Date	"2000/01/01"[Date - Publication] : "2024/01/01"[Date - Publication]
9. Combine	#7 AND #8
10 Exclude HPV	HPV OR "human papillomavirus"
11. Combine	#9 NOT #10
12. Exclude endometriosis	"Endometriosis"[MeSH] OR endometrios*
13. Combine	#11 NOT #12
14. Exclude diet	"Diet"[MeSH] OR diet*
15. Combine	#13 NOT #14
16 Study type	(Case Reports[Publication Type]) OR (Editorial[Publication Type]) OR (Clinical Trial[Publication Type])
17 Combine	#15 NOT #16
18 Reconstruction	Reconstruction[tiab]
19 Combine	#17 NOT #18
20 Infectious disease and contraception	"Communicable Diseases"[MeSH] OR "Infectious Disease Medicine"[MeSH] OR contraception[tiab]
21 Combine	#19 NOT #20

Search update 01.11.2024

1. Cancer diagnoses	oncolog*[tiab] OR cancer[tiab] OR neoplasm*[tiab]
2. Different age categories	adolescen*[tiab] OR teenag*[tiab] OR "young adult*"[tiab] OR "young peopl*"[tiab] OR AYA[tiab] OR TYA[tiab] OR “Young Adult”[MeSH] OR Adolescent[MeSH] OR “teenager*”[tiab] OR AYAs[tiab]
3. Combine	#1 AND #2
4. Set up in hospitals / clinics	"model* of care"[tiab] OR "cancer service*"[tiab] OR “cancer care”[tiab] OR "standard* of care"[tiab] OR "practice guideline"[tiab] OR “position paper”[tiab] OR “clinical care”[tiab] OR “distinctive characteristics”[tiab] OR “program development”[tiab] OR “specialised service*”[tiab] OR “specialized service*”[tiab] OR “clinical program*”[tiab]
5. Combine	#3 AND #4
6. Humans only	animals[Mesh] NOT humans[Mesh]
7. Combine	#5 NOT #6
8. Date	"2024/01/02"[Date - Publication] : "2024/11/01"[Date - Publication]
9. Combine	#7 AND #8
10 Exclude HPV	HPV OR “human papillomavirus”
11. Combine	#9 NOT #10
12. Exclude endometriosis	“Endometriosis”[MeSH] OR endometrios*
13. Combine	#11 NOT #12
14. Exclude diet	“Diet”[MeSH] OR diet*
15. Combine	#13 NOT #14
16 Study type	(Case Reports[Publication Type]) OR (Editorial[Publication Type]) OR (Clinical Trial[Publication Type])
17 Combine	#15 NOT #16
18 Reconstruction	Reconstruction[tiab]
19 Combine	#17 NOT #18
20 Infectious disease and contraception	“Communicable Diseases”[MeSH] OR “Infectious Disease Medicine”[MeSH] OR contraception[tiab]
21 Combine	#19 NOT #20
